# Whole-genome sequences of multidrug-resistant *Escherichia coli* in South-Kivu Province, Democratic Republic of Congo: characterization of phylogenomic changes, virulence and resistance genes

**DOI:** 10.1186/s12879-019-3763-3

**Published:** 2019-02-11

**Authors:** Leonid M. Irenge, Jerome Ambroise, Bertrand Bearzatto, Jean-François Durant, Raphaël B. Chirimwami, Jean-Luc Gala

**Affiliations:** 1 Center for Applied Molecular Technologies, Institute of Clinical and Experimental Research, Université catholique de Louvain, Clos chapelle-aux-champs, 30 B1.30.24, 1200 Brussels, Belgium; 2 Defence Laboratories Department, ACOS Ops&Trg, Belgian Armed Forces, Martelarenstraat, 181, 1800 Peutie, Belgium; 3 Université Catholique de Bukavu, P.O. Box 285, Bukavu, Democratic Republic of Congo

**Keywords:** *Escherichia coli*, ExPEC, Whole-genome sequencing, Multidrug resistance, Extended-spectrum beta-lactamases, Virulence

## Abstract

**Background:**

Extended-spectrum beta-lactamase (ESBL)-producing *Escherichia coli* are responsible for severe infections worldwide. Whereas their genotypic and pathogenic characteristics are not documented in Democratic Republic of Congo (DRC), recent studies conducted at the Bukavu General Hospital in the South Kivu province highlighted their high prevalence in extra-intestinal infections. Here we provide data on molecular characterization of ESBL producing-*Escherichia coli* isolates from patients with extra-intestinal infections at this provincial hospital.

**Methods:**

Whole-genome sequencing was carried out on 21 of these ESBL-producing Extra-intestinal Pathogenic *Escherichia coli* (ExPEC) for analysis of phylogenomic evolution, virulence factor and antimicrobial resistance (AMR) genes. Data were compared to phylogenetically close genomes using Multi-Locus Sequence Typing and Single Nucleotide Polymorphism-based phylogenetic approaches.

**Results:**

The distribution of *E. coli* sequence types (ST) was as follows: ST 131 (*n* = 7), ST405 (*n* = 4), ST410 (*n* = 2), and other STs (ST10, ST58, ST95, ST393, ST443, S617, ST648, and ST2450). All ST131 belonged to the O25b-ST131 pandemic clone. Unexpectedly, they harbored more virulence genes than their GenBank counterparts. IncF plasmid replicons included novel FIB 69, FII 105 and FII 107 alleles. ESBL-genes included the plasmid-mediated CTX-M-15 in all isolates, and the SHV-12 allele. Other AMR genes included blaOXA-1, blaTEM-1, as well as genes encoding resistance against aminoglycosides, quinolones, chloramphenicol, rifampicin, tetracyclines, sulfonamides and trimethoprim.

**Conclusion:**

Current data confirm the clonal spread of ESBL-producing ST131 and ST405 clones in patients from South Kivu, and the acquisition of resistance and virulence genes. A closer survey of AMR and virulence should therefore be prompted in this high-risk area.

**Electronic supplementary material:**

The online version of this article (10.1186/s12879-019-3763-3) contains supplementary material, which is available to authorized users.

## Background

**Fig. 1:**
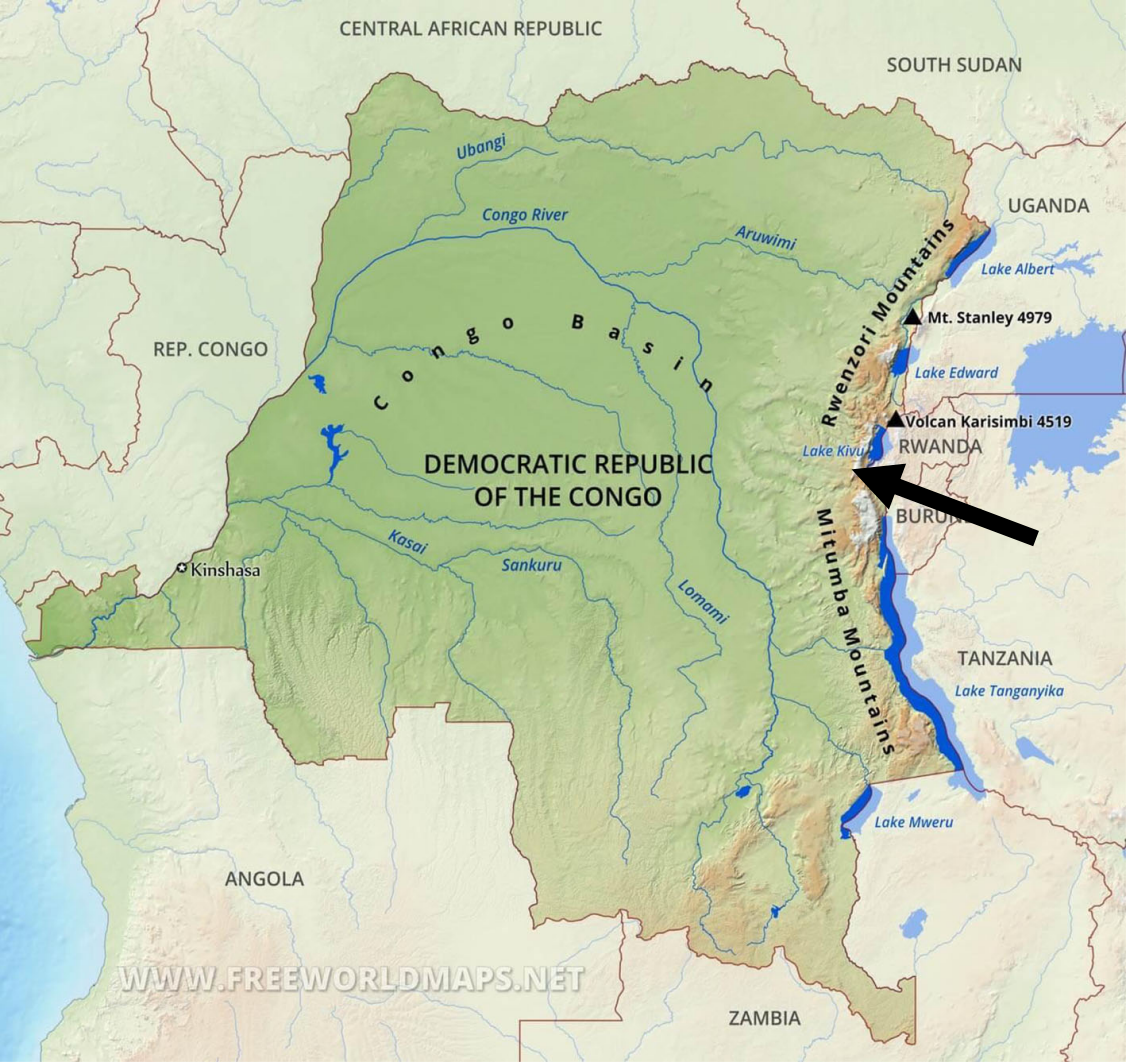
Map of the Democratic Republic of Congo. The area where the study was performed is indicated by a black arrow

The worldwide increasing prevalence of infections caused by multidrug-resistant (MDR) Gram-negative bacteria constitutes a serious threat to global public health, due to their association with a high morbidity and mortality rate which is fueled by the limited availability of effective antibiotics [1–3]. ESBL production is by far the most important determinant of rapid AMR spread among *Enterobacteriaceae* [4–6]. The dissemination of ESBL-producing *Enterobacteriaceae* is due to clonal expansion [7] and/or plasmid transfer [8]. ESBLs encoding genes are often located on large plasmids which are transferred to other bacteria by conjugation, enabling them to become ESBL producers [9, 10]. Beside ESBL genes, plasmids often harbor genes of resistance to multiple classes of antibiotics that result in MDR [8]. Several studies from all continents have consistently shown that CTX-M-15-producing *E. coli* is one of the most prevalent ESBL-producing *Enterobacteriaceae* [11] and that the global dissemination of ESBL-producing *E. coli* is associated with specific clones harboring a plasmid carrying the ESBL CTM-X-15 gene, especially ST131 and ST405 [12]. The same observation was made in a few African countries where CTX-M-15-producing *E. coli* belonging to phylogenetic groups A and D were found in extra-intestinal infections [13–18]. However, data on genotypic characterization of ExPEC are still lacking in several sub-Saharan countries among which DRC, the second largest African country [6]. This lack of accurate assessment of virulent and MDR ExPEC isolates makes it impossible to unravel the mechanisms underpinning their spread, hence to raise awareness about the best practices in health professionals. In two recent studies carried out at a tertiary care hospital in the Eastern province of South Kivu (Fig. 1), we observed a high prevalence of ESBL-producing *Enterobacteriaceae* in urinary tract and bloodstream infections among which a majority of ESBL-producing *E coli* in collected isolates [19, 20]. In the current study, a set of 21 ESBL-producing *E. coli* isolates were analyzed by whole-genome sequencing (WGS). This data set was used both to assess the phylogenomic relationship of MDR ESBL-producing *E. coli* isolates from DRC with GenBank genomes of MDR ESBL-producing ExPEC collected in other regions of the world, and to characterize their virulence and antimicrobial resistance genetic markers.

## Methods

### Bacterial strains

The set of 21 ESBL-producing *E. coli* isolates analyzed in the current study came from a collection of isolates assembled between 2012 and 2014 from inpatients at the Bukavu General Hospital, South Kivu (DRC, see Fig. 1). All isolates were phenotypically identified by standard biochemical tests including oxidase testing, triple sugar iron, imviC tests (Indole, Methyl Red, Voges-Proskaeur, and Citrate utilization), urease and motility.

### Antimicrobial susceptibility testing

The susceptibility to 14 antimicrobial agents (i.e. amikacin, amoxicillin, amoxicillin-clavulanic acid, ampicillin, ceftazidime, ceftriaxone, cefepime, cefotaxime, cefuroxime, chloramphenicol, ciprofloxacin, imipenem, trimethoprim-sulfamethoxazole and tetracycline) was determined by the disk diffusion method according to the European Committee on Antimicrobial Susceptibility Testing (EUCAST) guidelines as updated in January 2017 [21]. Minimum Inhibitory Concentrations (MIC) for each of the 15 antimicrobial agents was determined after 16–20 h incubation on Mueller-Hinton plates inoculated with suspensions of isolates at a fixed density (0.5 to 0.6 McFarland standard), using E-test strips (BioMérieux, Marcy l’Etoile, France) according to the manufacturer’s recommendations. Additionally, isolates were tested for ESBL-production by the double-disk synergy method on Mueller-Hinton agar using ceftazidime and ceftriaxone placed at a distance of 20 mm apart from a disk containing amoxicillin plus clavulanic acid. A clear-cut enhancement of the inhibition in front of either ceftazidime and/or ceftriaxone disks towards the clavulanic acid-containing disk (also called “champagne-cork” or “keyhole”) was interpreted as positive for ESBL production [22]. E-test strips (BioMérieux, Marcy l’Etoile, France) were used for confirmation of ESBL production, following the manufacturer’s instructions. *E. coli* ATCC 35218 and *Klebsiella pneumoniae* ATCC 700603 strains were used as ESBL-negative and positive controls, respectively. In addition, isolates were tested for the presence of the beta-lactamase AmpC phenotype using cefoxitin-cloxacillin disk diffusion test as described previously [23].

### Whole-genome sequencing

Whole-genome paired-end sequencing was performed using the MiSeq sequencer (Illumina, San Diego, CA, USA). Accordingly, genomic DNA (gDNA) from ESBL-producing *E. coli* was isolated using the EZ1 Advanced XL Biorobot and the tissue DNA kit (Qiagen, Hilden, Germany) with the Bacterial card, according to the manufacturer’s instructions. For each isolate, genomic DNA was quantified using Qubit® fluorometric quantitation (ThermoFisher Scientific, Oregon, USA) and normalized to 0.2 ng/μl. A standard Nextera XT library (Illumina, San Diego, USA) was constructed for each genome with 1 ng gDNA as recommended by the manufacturer. Briefly, gDNA was simultaneously fragmented and tagged with sequencing adapters in a single step using Nextera transposome (Nextera XT DNA Library Preparation Kit, Illumina, San Diego, USA). Tagmented DNA was then amplified (12-cycle PCR amplification) and cleaned up with AMPure beads. Nextera libraries were quantified using Qubit and the size profile was analyzed on 2100 Bioanalyzer using High sensitivity DNA assay kit (Agilent Technologies, Waldbronn, Germany). Fragments with size ranging from 828 to 1433 bases were generated. Libraries selected for sequencing were normalized to 1 nM and pooled. The 1 nM pooled library was denaturated and diluted prior to loading on a MiSeq paired-end 2 × 150 (MiSeq reagent kit V2 (300 cycles) or 2 × 300 base pairs (bp) (MiSeq reagent kit V3 (600 cycles) sequence run.

### Bioinformatics analysis

Paired-end reads from each *E. coli* isolate were assembled de novo using the Spades v.3.11.1 algorithm [24] to generate a draft genome sequence for each isolate and quality assessment for genome assemblies was carried out using QUAST 4.5 [25]. Raw genome data have been submitted to the European Nucleotide Archive (ENA, http://www.ebi.ac.uk/ena) and are available under accession number ERS1812814-ERS1812829. MLST typing was performed on draft (*n* = 21) and on complete genomes of ExPEC from GenBank by using the *E. coli* MLST scheme developed by Achtman [26] and the home-made Pathogenomic R package (https://github.com/JeromeAmbroise/Pathogenomics). The latter was used to screen all draft (n = 21) and complete genomes of *E. coli* sharing the same STs with DRC isolates for the virulence factor genes described in ExPEC [27–29] and/or available in the Virulence Finder database (https://cge.cbs.dtu.dk/services/VirulenceFinder/) with a threshold of 95% identity and a minimum length of 80%. Concurrently, each draft genome was screened for the presence of AMR genes. The complete list of screened genes was drawn up from the MEGARes database (https://megares.meglab.org). In order to selectively identify AMR genes acquired through horizontal gene transfer, the list based on MEGARes data was restricted to genes that were also found in the ResFinder database (https://cge.cbs.dtu.dk/services/ResFinder/), using BLASTn. In addition, SNP-based AMR chromosomal determinants were identified using the ARIBA software [30] with the MEGARes database. Assembled contigs were further assessed for the presence of plasmid replicons using the plasmid multilocus sequence typing (pMLST) database [31]. The F plasmids were further categorized by the FAB (FII, FIA, FIB) formula using the replicon sequence typing (RST) scheme described by Villa [32]. The DNA sequences of novel FIB and FII replicons were submitted to the pMLST database curator (https://pubmlst.org/plasmid/) for the assignment of the ST. All drafts (*n* = 21) and ST-relevant complete genomes from GenBank were submitted to kSNP3.0 for SNP identification and Maximum Likelihood phylogenetic tree construction. This software performs SNP identification without genome alignment nor requirement for reference genomes. In parallel, WGS data were used to characterize *E. coli* isolates through the combination of four DNA gene markers (i.e. ArpA, chuA, yjaA and TSPE4-C2) as described by Clermont et al. [33]. In brief, *E. coli* draft genomes were screened for the presence of these four genetic markers, a combination thereof determining the phylogenetic clustered distribution of the isolates. Those isolates which belong to the B2 phylogenetic group were further screened for the ST131-O25b clone-specific silent SNPs in the *E. coli pab*B gene (C267T and G573A, accession number: CP015085) as previously described [34].

## Results

### Antimicrobial susceptibility patterns

**Fig. 2:**
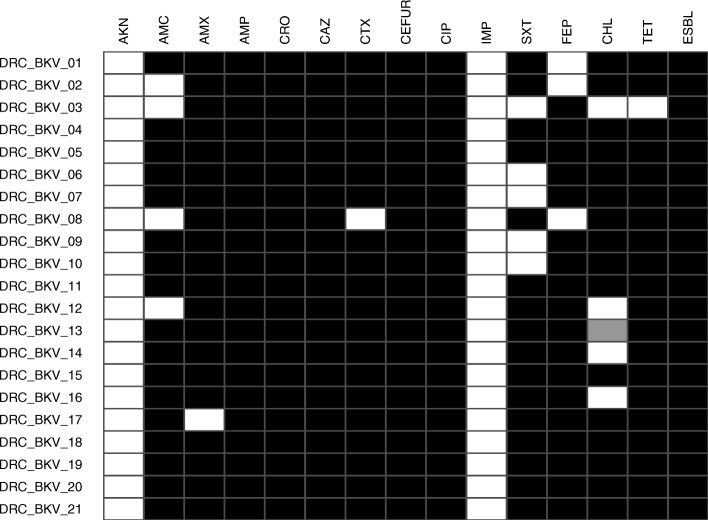
Heatmap of antimicrobial susceptibility profiles of DRC ESBL-producing ExPEC isolates. White, grey and black boxes: susceptible, intermediate and resistant to the corresponding antimicrobial drug. All DRC ESBL-producing ExPEC isolates retained susceptibility to imipenem, while displaying resistance to at least three of the following antimicrobial families: beta-lactams (including third and fourth generation cephalosporins), quinolones, sulfonamids, tetracycline and chloramphenicol. AKN: amikacin; AMX: amoxicillin; AMC: amoxicillin-clavulanic acid; AMP: ampicillin; CRO: ceftriaxone; CAZ: ceftazidime; CTX: cefotaxime; CEFUR: cefuroxime; CIP: ciprofloxacin; IMP: imipenem; SXT: trimethoprim-sulfamethoxazole; FEP: cefepime; CHL: chloramphenicol; TET: tetracycline, ESBL: Extended Spectrum Beta-Lactamase phenotype

All isolates (*n* = 21) were MDR ESBL-producing *E. coli* (Fig. 2). MIC data are provided in in Table 1. The majority of isolates displayed low susceptibility to amoxicillin, amoxicillin-clavulanic acid, ampicillin, ceftazidime, cefuroxime, ceftriaxone, cefotaxime, chloramphenicol, ciprofloxacin, imipenem, trimethoprim-sulfamethoxazole and tetracycline. In contrast, all and 19/21 DRC *E. coli* isolates were susceptible to imipenem and amikacin, respectively.

**Table 1:** MIC values (mg/L) of antimicrobial agents for the 21 MDR *E. coli* from DRC isolates were deemed as Susceptible, Intermediate or Resistant to antibiotics based on breakpoints values provided by EUCAST. Abbreviations are the same as for Fig. 2, with the additional abbreviations CAZ + CLAV: ceftazidime-clavulanic acid; CTX + CLAV: cefotaxime-clavulanic acid

	AKN	AMX	AMC	AMP	CAZ	CAZ + CLAV	CRO	CTX	CTX + CLAV	FEP	CTX	CEFUR	CHL	CIP	IMP	SXT	TET
DRC_BKV_01	4	> 256	16	> 256	6	0.094	12	> 16	0.094	6	12	> 256	8	> 32	0.125	> 32	> 256
DRC_BKV_02	4	> 256	8	> 256	4	0.064	16	> 16	0.032	6	8	> 256	> 256	> 32	0.064	> 32	> 256
DRC_BKV_03	2	> 256	8	> 256	> 32	0.064	> 256	> 16	0.125	> 256	> 32	> 256	0.75	8	0.125	0.032	0.38
DRC_BKV_04	16	> 256	12	> 256	12	0.125	> 256	> 16	0.094	12	> 32	> 256	> 256	> 32	0.094	> 32	> 256
DRC_BKV_05	12	> 256	16	> 256	16	0.25	> 256	> 16	0.094	12	> 32	> 256	> 256	> 32	0.125	> 32	> 256
DRC_BKV_06	3	> 256	12	> 256	> 32	0.25	> 256	> 16	0.38	48	> 32	> 256	> 256	> 32	0.125	0.25	> 256
DRC_BKV_08	2	> 256	8	> 256	8	0.064	8	8	0.032	0.064	1	64	> 256	> 32	0.125	> 32	> 256
DRC_BKV_07	1.5	> 256	12	> 256	> 32	0.25	> 256	> 16	0.5	48	> 32	> 256	> 256	> 32	0.125	0.125	> 256
DRC_BKV_09	2	> 256	12	> 256	> 32	0.125	> 256	> 16	0.125	48	> 32	> 256	> 256	> 32	0.064	0.125	> 256
DRC_BKV_10	1.5	> 256	12	> 256	> 32	0.25	> 256	> 16	0.25	64	> 32	> 256	> 256	> 32	0.125	0.125	> 256
DRC_BKV_11	3	> 256	12	> 256	8	0.064	> 256	> 16	0.047	6	> 32	> 256	> 256	16	0.125	> 32	128
DRC_BKV_12	4	> 256	8	> 256	> 32	0.19	> 256	> 16	0.19	> 256	> 32	> 256	4	> 32	0.125	> 32	> 256
DRC_BKV_13	6	> 256	16	> 256	> 32	0.125	> 256	> 16	0.094	64	> 32	> 256	4	> 32	0.125	> 32	> 256
DRC_BKV_14	4	> 256	16	> 256	> 32	0.125	> 256	> 16	0.094	64	> 32	> 256	3	> 32	0.125	> 32	96
DRC_BKV_15	2	> 256	16	> 256	32	0.19	> 256	> 16	0.094	24	> 32	> 256	> 256	> 32	0.125	> 32	> 256
DRC_BKV_16	3	> 256	12	> 256	12	0.125	> 256	> 16	0.064	24	> 32	> 256	4	> 32	0.125	> 32	3
DRC_BKV_17	6	0.5	8	> 256	> 32	> 4	> 256	> 16	> 1	> 256	> 32	> 256	> 256	> 32	0.125	> 32	> 256
DRC_BKV_18	2	> 256	12	> 256	> 16	0.19	> 256	> 32	0.125	192	> 32	> 256	> 256	> 32	0.125	> 32	> 256
DRC_BKV_19	4	> 256	12	> 256	8	0.064	> 256	> 16	0.032	16	> 32	> 256	> 256	12	0.125	> 32	64
DRC_BKV_20	6	> 256	12	> 256	> 32	0.125	> 256	> 16	0.032	64	> 32	> 256	> 256	> 32	0.125	> 32	> 256
DRC_BKV_21	4	> 256	16	> 256	32	0.125	> 256	12	0.125	24	> 32	> 256	> 256	> 32	0.125	> 32	> 256

### Whole-genome sequencing and assembly

Computation of the total number of reads and quality metrics of the assemblies (Additional file 1) showed homogenous results with a good quality profile for all isolates.

### Phylogenomic analysis

**Fig. 3:**
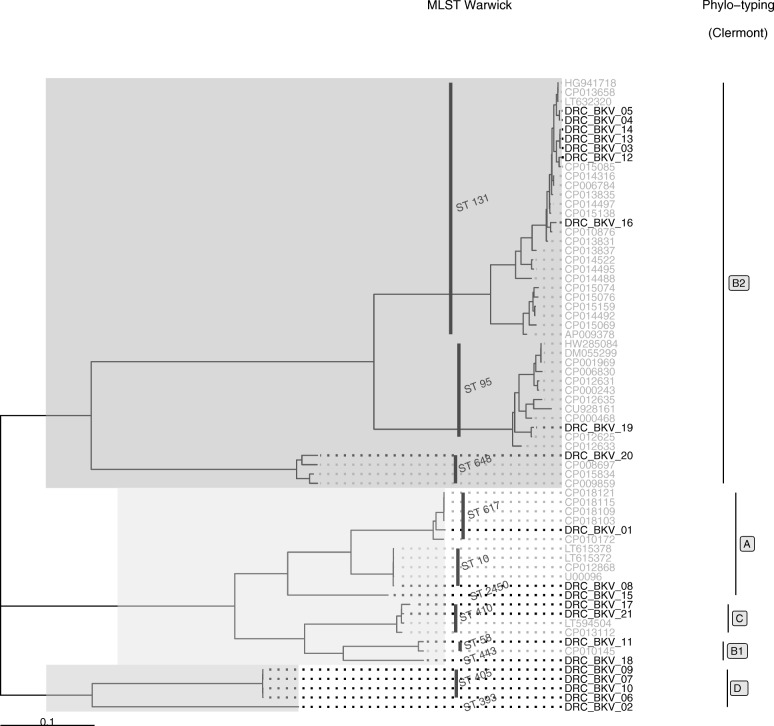
Maximum Likelihood phylogenetic tree based on the SNP differences within the core genomes of DRC ESBL-producing ExPEC (in black) along with other well characterized counterparts from GenBank (in grey)

*E. coli* isolates (n = 21) were clustered into three major clades (Fig. 3). The first one grouped seven ST131 ExPEC isolates (DRC_BKV_03, DRC_BKV_04, DRC_BKV_05, DRC_BKV_12, DRC_BKV_13, DRC_BKV_14, and DRC_BKV_16), one ST95 isolate (DRC_BKV_19), and one ST648 isolate (DRC_BKV_20). All ST131 isolates belonged to the same sub-clade and to the phylogenetic group B2 according to Clermont et al. [33]. All displayed the C267T and the G573A substitutions in the *pab*B gene in line with their O25b-ST131 status [34] (Fig. 3). The second clade included seven isolates belonging to unrelated various STs (i.e., DRC_BKV_01: ST617; DRC_BKV_08: ST10; DRC_BKV_15: ST2450; DRC_BKV_17 and DRC_BKV_21: ST410; DRC_BKV_11: ST58; DRC_BKV_18: ST443).

The third clade contained four ST405 isolates (i.e., DRC_BKV_06, DRC_BKV_07, DRC_BKV_09, and DRC_BKV_10), and one ST393 isolate (DRC_BKV_02).

### Detection of virulence factors genes

**Fig. 4:**
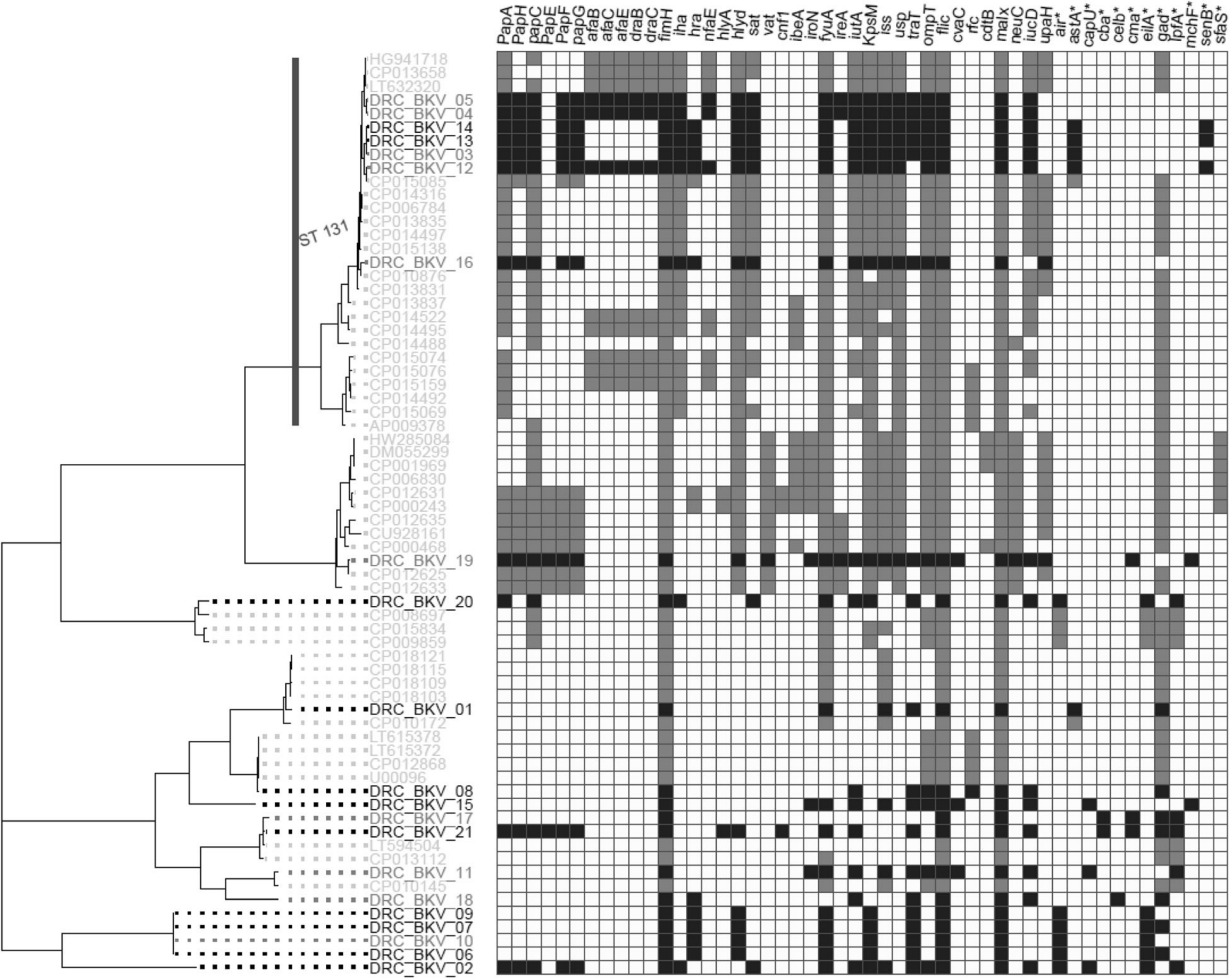
Virulence genes identified both in DRC ESBL-producing ExPEC (in dark grey for *E. coli* isolated from bloodstream, and in black for those isolated from urine samples), and in ExPEC from GenBank (in light grey). A gene was deemed present if it matched the reference sequence (minimal identity match of 95% with a minimal coverage of 80% of the gene sequence). * Virulence factor genes not extensively characterized in ExPEC as per previous reports [27–29], but described and/or available in the Virulence Finder database https://cge.cbs.dtu.dk/services/VirulenceFinder/)

At first sight, virulence factors identified in DRC ST131 *E.coli* isolates are similar to those reported in pandemic CTX-M-15-producing *E. coli* O25b-ST131 [28, 35–37]. However, *E. coli* O25-b-ST131 from DRC harbored significantly (*p* < 0.01, t-test) more virulence genes (Fig. 4) as illustrated by the presence of the *tra*T gene carried by all but one (DRC_BKV_12) DRC *E.coli* O25b-ST131. This gene was not detected in any of the *E. coli* genomes selected from Genbank (Fig. 4). Likewise, two out of 7 DRC O25b-ST131 isolates (i.e., DRC_BKV_04 and DRC_BKV_05) harbored the *ire*A virulence gene, which was absent from similar strain sequences in GenBank [35]. DRC ST131 *E. coli* were mostly (5/7) isolated from bloodstream and, as expected, harbored more virulence genes than DRC ST405 *E. coli*. The latter isolates were mostly (3/4) isolated from urine.

### Detection of AMR genes

**Fig. 5:**
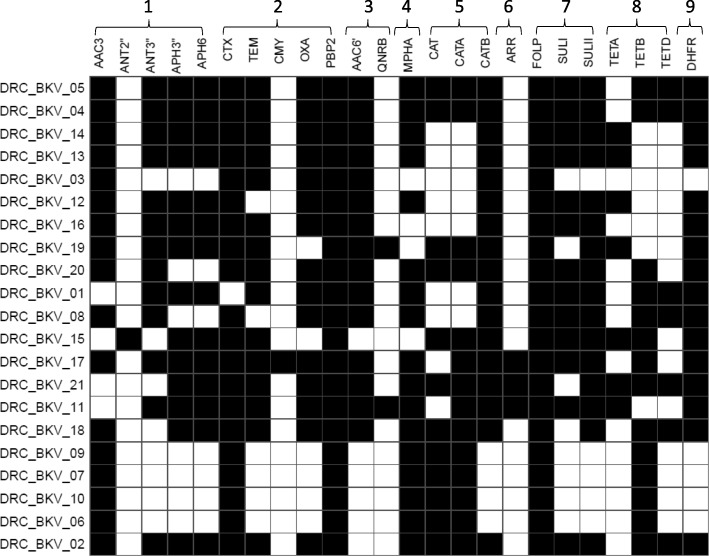
Heatmap showing AMR genes found in the draft genome of DRC ESBL-producing ExPEC (*n* = 21) and present both in MEGARes and ResFinder. AMR genes are annotated as in MEGARes. The names of the strains indicated on the y axis are presented in the same order as in Figs. 2 and 3. 1. *AMR genes for aminoglycosides:* AAC3: Aminoglycoside N acetyltransferases AAC3; “ANT2”: Aminoglycoside O nucleotidyltransferases ANT2 DPRIME; “ANT3”: Aminoglycoside O nucleotidyltransferases ANT3 DPRIME; “APH3”: Aminoglycoside O phosphotransferases APH3-DPRIME; APH6: Aminoglycoside O phosphotransferases. 2. *AMR genes for beta-lactams:* CTX: Class A beta-lactamases CTX (in our study, all CTX genes consisted of CTX-M-15); TEM: Class A beta-lactamases TEM; CMY: Class C beta-lactamases CMY; OXA: Class D beta-lactamases OXA; PBP2: Penicillin binding protein PBP2. 3. A*MR genes for quinolones:* AAC(6′): Aminoglycoside N acetyltransferase (corresponds to AAC(6′)-Ib-cr-1*in all DRC E. coli isolates); QNRB: Quinolone resistance protein Qnr QNRB. 4. AMR gene for macrolides:* MPHA: Macrolide phosphotransferase MPHA. 5. *AMR genes for phenicols:* Chloramphenicol acetyltransferase CAT; Chloramphenicol acetyltransferases CATA; Chloramphenicol acetyltransferase CATB. 6. AMR genes for rifampicin: ARR: Rifampicin ADP ribosyltransferase Arr ARR. 7. *AMR genes for sulfonamides:* FOLP: Sulfonamide resistant dihydropteroate synthases FOLP; SULI: Sulfonamide resistant dihydropteroate synthases SULI; SULII: Sulfonamide resistant dihydropteroate synthases SULII. 8. *AMR genes for tetracyclines:* TETA: Tetracycline resistance major facilitator superfamily MFS efflux pumps TETA; TETB: Tetracycline resistance major facilitator superfamily MFS efflux pumps TETB; TETD: Tetracycline resistance major facilitator superfamily MFS efflux pumps TETD. 9. A*MR gene trimethoprim:* DHFR: Dihydrofolate reductase DHFR. It is of note that DRC_BKV_01 contained a “CTX-M” sequence as annotated in the MEGARes database. The BLAST analysis confirmed however that this annotation corresponded rather to the *tnp*A gene and not to CTX-M. DRC_BKV_01 is therefore reported as CTX-free in the figure

Each draft genome sequence of ExPEC isolates from South Kivu harbored AMR genes. They consisted in chromosomal SNP-based determinants of AMR and/or plasmid-mediated AMR to various classes of antibiotics (Additional file 2 and Fig. 5). Some chromosomal SNP-based determinants of AMR corresponded to amino acid substitutions leading to resistance to several antibiotics, e.g. quinolones, sulfonamides, rifampicin, and elfamycins. Other chromosomal SNP-based determinants of AMR caused amino acids substitutions in several MDR genes (OMPF porin, PhoP multi-drug efflux pump) [38] and/or in genes which regulate the expression of several AMR genes, such as MARR (Multiple Antibiotic Resistance Regulator) and soxS (a member of Superoxide regulon) [39]. Analysis of MIC values for ciprofloxacin revealed that, whereas all DRC *E. coli* isolates were resistant to ciprofloxacin, high level resistance to this drug was overall associated with amino acid substitutions in quinolone-resistance-determining-regions (QRDR) of gyrA gene (S83 L, D87N) and/or in QRDR of parC gene (S80I, E84V, S57T, E84G). In addition, several SNPs resulting in amino acid substitutions were also characterized in gyrB and parE genes. Noticeably, none of these substitutions occurred in the respective QRDRs of both latter genes. These findings are consistent with other studies emphasizing the importance of substitutions in QRDRs of gyrA and parC proteins in the emergence of high level resistance to quinolones [40, 41]. However, given the limited set of data analyzed in this study, a confirmation of the role played by chromosomal SNP-based determinants in the emergence of quinolone resistance in DRC isolates requires further assessment.

The CTX-M-15 gene, which encodes a protein responsible for the ESBL phenotype, was detected in all but one isolate (DRC_BKV_01). WGS-based analysis identified plasmid replicons in 21/21 isolates. Beside well-characterized alleles, RST revealed the presence of three new plasmid replicons, FIB 69 FII 105 and FII 107, which are reported here for the first time. Twelve different RST profiles were characterized in the 22 plasmid replicons: F105:A1:B69 (DRC_BKV_06, DRC_BKV_07, DRC_BKV_09, and DRC_BKV_10), F31:A4:B1 (DRC_BKV_01, DRC_BKV_08, and DRC_BKV_16), F48:A1:B49 (DRC_BKV_04 and DRC_BKV_05), F1:A2:B20 (DRC_BKV_13 and DRC_BKV_14), F2:A-:B1 (DRC_BKV_11 and DRC_BKV_15), F1:A1:B1 (DRC_BKV_18 and DRC_BKV_20), F1:A1:B16 (DRC_BKV_12), F36:A1:B1 (DRC_BKV_02), F1:A1:B49 (DRC_BKV_17), F107:A-:B:1 (DRC_BKV_19), F2:A1:B1 (DRC_BKV_21), and F1:A:2:B- (DRC_BKV_03). It is noteworthy that, except for IncF, no other incompatibility plasmid replicon types (i.e., IncA/C, IncH1, IncH2, IncI1, and IncN) were identified in these DRC isolates.

## Discussion

WGS was used to analyze non-duplicated ESBL-producing *E. coli* isolates (*n* = 21) collected from patients at a tertiary care hospital in South-Kivu province of the DRC between 2014 and 2016. Despite the limited sample size, this study provides the first evidence that pandemic ESBL-producing *E. coli* O25b-ST131 and ST405 carrying blaCTX-M-15 are present in this DRC province, a factor that may be driving their widespread dissemination. Whereas establishing clonality between isolates sharing the same ST and high sequence similarity is difficult [37], data analysis of core genome, virulence and AMR genes supports the assumption that some of these isolates may have diverged recently from a common ancestor. The hypothesis of a common O25b-ST131 ancestor is supported by the perfect match between virulence and AMR genes as well as RST profiles of DRC_BKV_04 and DRC_BKV_05, with as few as 7 SNPs distinguishing their draft genome. It also applies to DRC_BKV_13 and DRC_BKV_14 whose core genomes only differed at 4 SNPs, as well as to the ST405 sub-clade (DRC_BKV_06, DRC_BKV_07, DRC_BKV_09 and DRC_BKV_10) which differed only by 1 SNP. These observations strengthen the hypothesis that local O25b-ST131 and ST405 sub-clades diverged recently from common ancestors.

Conversely, a markedly different virulence pattern rules out a clonal relationship between DRC_BKV_12 and Saudi *E. coli* isolate (accession n° CP015085) despite a close relatedness of their core genomes. Likewise, ST131 isolates from DRC do not seem to be closely related to other well characterized international isolates (i.e., NCTC13441: accession n° LT632320; uk_P46212: accession n° CP013658; EC958: accession n° HG941718) given their dissimilar virulence gene patterns.

Current data illustrate that ExPEC isolates from Bukavu, probably because of a permanent selective pressure of antibiotics, undergo a continuous remodeling process leading to spontaneous SNPs mutations and acquisition of virulence and AMR genes. This process may generate a genetic drift and/or shift from a common ancestor and the subsequent emergence of new clones. It is of note that *E. coli* belonging to other phylogenetic groups (A, B1) have also been isolated in extra-intestinal infections in other African countries [14, 15], but not yet in Western countries [27, 28].

To date, no convincing explanation for these discrepant observations can be put forward. It is however noteworthy that this study did not assess the clinical history of patients with infections caused by ESBL-producing bacteria, notably the effect of immune response due to HIV, malnutrition or other debilitating diseases. However, whether this may pave the way to severe ExPEC infections with non-B2 and non-D ESBL-producing *E. coli* requires confirmation.

Current WGS-based genotyping results corroborate our previous observations with ESBL-producing *Enterobacteriaceae* in urinary and bloodstream isolates in the South Kivu province [19, 20]. Extended set of virulence and AMR genes is expected to provide ESBL-producing *E. coli* strains capacity for surviving and thriving in their host and surrounding environment in presence of several antimicrobial agents [42]. The characterization of novel beta-lactamases and replicons suggest a high level of genetic plasticity within ExPEC plasmids carrying AMR genes. Moreover, the current irrational use of antibiotics in DRC is expected to facilitate nosocomial and community transmission and uncontrolled spread of these ESBL-producing ExPEC isolates.

## Conclusions

Our results show that resistance of ESBL-producing *E. coli* to multiple classes of antibiotics in South Kivu Province of DRC is driven by several CTX-M-15 producing ST among which ST131 and ST405, as well as other STs considered not to be associated with ExPEC infections. These results corroborate previous observations on the staggering ability of pandemic clones O25b-ST131 and ST405 to adapt to new environmental conditions while also highlighting the continuous accumulation of both virulence and AMR genes in these pathogens. A vigorous approach through regional and international cooperation is needed to mitigate what looks like the inexorable spread of ESBL-producing *E. coli* in South Kivu province and beyond.

## Additional files

Additional file 1: Quality metrics of genome assemblies (QUAST 4.5). (XLS 36 kb)

Additional file 2: Amino acid substitutions in chromosomal AMR genes, as identified using ARIBA software. Genes involved in quinolones AMR (gyrA, gyrB, parC and parE) are highlighted in grey. Amino acid substitutions in quinolone resistance-determining region (QRDR) of the above-cited genes are in bold. (XLS 41.5 kb)

## Abbreviations

AMR: Antimicrobial resistance
DRC: Democratic Republic of Congo
ESBL: Extended-spectrum beta-lactamase
EUCAST: European Committee on Antimicrobial Susceptibility Testing
ExPEC: Extra-intestinal Pathogenic *Escherichia coli*
gDNA: Genomic DNA
MDR: Multidrug-resistant
MIC: Minimum Inhibitory Concentrations
MLST: Multi-Locus Sequence Typing
SNP: Single Nucleotide Polymorphism
ST: Sequence Type
WGS: Whole-genome sequencing
